# Identification of Potential Crucial Genes and Key Pathways in Breast Cancer Using Bioinformatic Analysis

**DOI:** 10.3389/fgene.2019.00695

**Published:** 2019-08-02

**Authors:** Jun-Li Deng, Yun-hua Xu, Guo Wang

**Affiliations:** ^1^Department of Clinical Pharmacology, Xiangya Hospital, Central South University, Changsha, China; ^2^Institute of Clinical Pharmacology, Central South University, Hunan Key Laboratory of Pharmacogenetics, Changsha, China; ^3^Engineering Research Center of Applied Technology of Pharmacogenomics, Ministry of Education, Changsha, China; ^4^National Clinical Research Center for Geriatric Disorders, Changsha, China

**Keywords:** breast cancer, GEO, TCGA, differentially expressed genes, bioinformatics, survival, biomarker

## Abstract

**Background:** The molecular mechanism of tumorigenesis remains to be fully understood in breast cancer. It is urgently required to identify genes that are associated with breast cancer development and prognosis and to elucidate the underlying molecular mechanisms. In the present study, we aimed to identify potential pathogenic and prognostic differentially expressed genes (DEGs) in breast adenocarcinoma through bioinformatic analysis of public datasets.

**Methods:** Four datasets (GSE21422, GSE29431, GSE42568, and GSE61304) from Gene Expression Omnibus (GEO) and the Cancer Genome Atlas (TCGA) dataset were used for the bioinformatic analysis. DEGs were identified using LIMMA Package of R. The GO (Gene Ontology) and KEGG (Kyoto Encyclopedia of Genes and Genomes) analyses were conducted through FunRich. The protein-protein interaction (PPI) network of the DEGs was established through STRING (Search Tool for the Retrieval of Interacting Genes database) website, visualized by Cytoscape and further analyzed by Molecular Complex Detection (MCODE). UALCAN and Kaplan–Meier (KM) plotter were employed to analyze the expression levels and prognostic values of hub genes. The expression levels of the hub genes were also validated in clinical samples from breast cancer patients. In addition, the gene-drug interaction network was constructed using Comparative Toxicogenomics Database (CTD).

**Results:** In total, 203 up-regulated and 118 down-regulated DEGs were identified. Mitotic cell cycle and epithelial-to-mesenchymal transition pathway were the major enriched pathways for the up-regulated and down-regulated genes, respectively. The PPI network was constructed with 314 nodes and 1,810 interactions, and two significant modules are selected. The most significant enriched pathway in module 1 was the mitotic cell cycle. Moreover, six hub genes were selected and validated in clinical sample for further analysis owing to the high degree of connectivity, including CDK1, CCNA2, TOP2A, CCNB1, KIF11, and MELK, and they were all correlated to worse overall survival (OS) in breast cancer.

**Conclusion:** These results revealed that mitotic cell cycle and epithelial-to-mesenchymal transition pathway could be potential pathways accounting for the progression in breast cancer, and CDK1, CCNA2, TOP2A, CCNB1, KIF11, and MELK may be potential crucial genes. Further, it could be utilized as new biomarkers for prognosis and potential new targets for drug synthesis of breast cancer.

## Introduction

Breast cancer is the most common non-cutaneous malignancy in American women, which has an estimated 268,600 new cases and 41,760 deaths in 2019, representing 30% of all new cancer cases and 15% of cancer-related deaths (Gradishar et al., 2018; Siegel et al., 2019). In China, an increasing trend in cancer-related mortality has been observed for 3 out of the 10 most common cancers, breast, ovary, and cervical cancer, while it seems to be steady over the years for other cancers such as lung, colorectal, uterine, and thyroid cancer (Chen, 2015).

Like most cancers, breast cancer is categorized to different types based on the difference of molecular characteristics, histopathological appearance, and clinical outcome. Based on the molecular classifications, breast cancer can be mainly divided into six subgroups: normal-like, luminal A and B, HER2-positive, basal-like, and claudin-low. Basal-like and claudin-low subtypes, characterized by the lack of estrogen receptor (ER), progesterone receptor (PR), and HER2 expression, belong to the type of triple-negative breast cancer (TNBC) and have a greater possibility of distant disease recurrence and a high frequency of visceral metastases (Kast et al., 2015). A recent meta-analysis with a large cohort of TNBC cases has subclassified TNBC into at least four subtypes: basal-like immune-activated (BLIA), basal-like immune-suppressed (BLIS), luminal androgen receptor (LAR), and mesenchymal (MES) tumor (Lehmann et al., 2011; Burstein et al., 2015). This subclassification is further supported by the Cancer Genome Atlas (TCGA) Program through the analysis of mRNA, miRNA, DNA, and epigenetic profiles (Cancer Genome Atlas Network, 2012). Each subgroup of breast cancer adopts a different therapeutic regimen and a specific prognosis. However, accumulating evidences have supported the hypothesis that these breast cancer subgroups share similar activated or repressed genes and common signaling pathways (Adamo et al., 2011; Dey et al., 2017). These genes and signaling pathways are probably implicated in the tumorigenesis and progression of breast cancer.

Genome-wide molecular profiling is able to reveal molecular changes in tumorigenesis and progression and has proved to be a high-efficient way to identify key genes (Gao et al., 2016; Li et al., 2016; Zhu et al., 2017). In the current study, we aim to investigate the potential crucial genes and key pathways in breast cancer tumorigenesis and prognosis through bioinformatic analysis of gene expression profiling and clinical characteristics in public datasets. The obtained data indicated that some hub genes were associated with breast cancer tumorigenesis and prognosis.

## Materials and Methods

### Breast Adenocarcinoma Datasets

Four independent breast adenocarcinoma gene expression profiles (GSE21422, GSE29431, GSE42568, and GSE61304), which were composed of 235 primary breast tumor samples and 38 normal breast tissue samples, were downloaded from the Gene Expression Omnibus (GEO) database (https://www.ncbi.nlm.nih.gov/geo/) and exploited as discovery datasets to identify DEGs. All of these datasets were obtained from the microarray platform of Affymetrix Human Genome U133 Plus 2.0 Array [HG-U133_Plus_2]. Furthermore, as the gene expression profiling and clinical information of patients were available, 1,105 breast cancerous and 113 non-cancerous samples were selected from TCGA (http://tcga-data.nci.nih.gov) and used as a validation dataset. Samples with incomplete information were removed before analysis. Detailed information of datasets was listed in **Table 1**.

**Table 1:** Characteristics of datasets in this study.

Dataset	Platform	Sample		Tumor type	Country
		Normal	Tumor		
GSE21422	Affymetrix HG-U133_Plus_2	5	14	Breast cancer	Germany
GSE29431	Affymetrix HG-U133_Plus_2	12	54	Breast cancer	Spain
GSE42568	Affymetrix HG-U133_Plus_2	17	109	Breast cancer	Ireland
GSE61304	Affymetrix HG-U133_Plus_2	4	58	Breast cancer	Singapore
TCGA	IlluminaHiSeq	113	1,105	Breast cancer	USA

### Data Preprocessing

The analysis of raw probe-level data (.CEL files) was performed using the robust multiarray average algorithm RMA in the Affy package of R (Irizarry et al., 2003) after background correction and quantile normalization, and the expression values were then obtained. The averages of the probe sets of values were calculated as the expression values for the same gene with multiple probe sets (Li et al., 2014).

### Identification of DEGs

Identification of DEGs was performed using the LIMMA package of R (Diboun et al., 2006). The adjusted *P*-values (adj *P*-value) were adopted to avoid the occurrence of false-positive results. Genes with |log2 ^fold change (FC)^| larger than 1 and adj *P*-value < 0.01 were taken as differentially expressed genes between tumors and normal tissues. Ggplot2 and VennDiagram packages of R were applied to generate volcano plot and Venn diagram, respectively, for the visualization of the identified DEGs.

### Functional Enrichment Analysis

FunRich is a software used mainly for gene functional classification that provides a comprehensive set of functional annotation for researchers to understand biological characteristics (Pathan et al., 2017). GO (Gene Ontology) function and KEGG (Kyoto Encyclopedia of Genes and Genomes) pathway enrichment analyses of the DEGs were performed through FunRich.

### PPI Network Construction and Module Analysis

The Search Tool for the Retrieval of Interacting Genes (STRING; http://string.embl.de/) is a biological database designed to construct a PPI network of DEGs based on the known and predicted PPIs, and then analyze the functional interactions between proteins (Szklarczyk et al., 2015). Based on the STRING online tool, PPIs of the DEGs were constructed with a confidence score ≥ 0.7. Subsequently, the PPI network was visualized by means of Cytoscape software (version 3.5.1). Furthermore, the plug-in of Molecular Complex Detection (MCODE) (Bader and Hogue, 2003) in Cytoscape software was applied to explore the significant modules in PPI network. The advanced options set as degree cutoff = 2, K-Core = 2, and Node Score Cutoff = 0.2. Subsequently, the enrichment analysis of DEGs in module 1 and module 2 was carried out using FunRich and visualized by R software.

### Survival Analysis of Hub Genes

The Kaplan–Meier plotter (http://kmplot.com/analysis/) could assess the effect of 54,675 genes on survival using 18,674 cancer samples (Lanczky et al., 2016). These cover 5,143 breast, 1,816 ovarian, 2,437 lung, 1,065 gastric, and 364 liver cancer patients with relapse-free and overall survival information, which were mainly based on database of GEO, TCGA, and EGA. The aim of the tool is a meta-analysis based on biomarker assessment to have a benefit in clinical decisions, health care policies, and resource allocation (Lacny et al., 2018). In our study, we analyzed the overall survival of individual hub genes through the Kaplan–Meier plotter in breast cancer. Patients were classified into two groups according to the median of each hub gene expression in Kaplan–Meier plotter for overall survival. This classification method could show the survival probability differences between high-expression group and low-expression group.

### Expression Analysis of Hub Genes

The analysis of relative expression of the six hub genes was performed using UALCAN (http://ualcan.path.uab.edu), a user-friendly, interactive web resource for analyzing cancer transcriptome data (TCGA and MET500 transcriptome sequencing) (Chandrashekar et al., 2017). UALCAN allows users to identify biomarkers or to perform *in silico* validation of potential genes of interest. One of the portal’s user-friendly features is that it allows analysis of relative expression of a query gene(s) across tumor and normal samples, as well as in various tumor molecular subtypes such as individual age, gender, tumor stages, or other clinicopathological features. Therefore, we explored the relative expression of six hub genes *via* UALCAN based on various tumor molecular subtypes and clinicopathological features of breast cancer.

### Hub Gene-Drug Interaction Network Analysis

The hub gene-drug interaction network was constructed using Comparative Toxicogenomics Database (CTD) (Davis et al., 2013) for chemotherapeutic drugs that could reduce or elevate the mRNA or protein expression levels of the hub genes. Briefly, these hub genes of CDK1, CCNA2, TOP2A, CCNB1, KIF11, and MELK were searched in CTD database, and the hub gene-drug interaction networks were visualized by using Cytoscape version 3.5.1.

### Patients and Tissue Samples

All 22 breast cancer tissues and paired adjacent non-cancerous tissues were obtained from patients who had undergone radical surgical resection of breast cancer from March 2015 to December 2016 at Xiangya Hospital of Central South University, China. The paired adjacent non-cancerous tissues were dissected by the surgeons 5-cm away from the tumor edge. Tissue samples were stored at liquid nitrogen until total RNA was extracted. These breast cancers patients were diagnosed and graded by the pathological features in the Department of Pathology, Xiangya Hospital. This study was approved by the Ethics Committee of Xiangya Hospital, and the informed consent forms (IFC) were obtained from all the patients.

### Quantitative Real-Time RT-PCR

Quantitative real-time RT-PCR were carried out as previously described with minor modifications (Sun et al., 2015). Brieﬂy, total RNA of 1 µg was reversely transcribed in a 20 μl reaction using the PrimeScript^™^ RT Reagent Kit with gDNA Eraser (Takara, Dalian, China, code no: RR047A) according to the manufacturer’s protocol. The reaction products were then diluted with 80 μl distilled water. The real-time PCR reaction was composed of 2 μl of diluted reverse transcription product, 10 µl of 2X SYBR^®^ Premix DimerEraser^™^ (Takara Bio Inc., code no: RR091A) and 0.6 µl of forward and reverse primers (0.3 μM). The reaction was performed in a Light Cycler@ 480 II Sequence Detection System (Roche, Basel, Switzerland) for 40 cycles (95°C for 5 s, 55°C for 30 s, 72°C for 30 s) after an initial 30 s denaturation at 95°C. β-Actin was used as an internal control. The RNA levels of tumor samples and paired adjacent samples were calculated using the 2^−ΔCt^ method. All primers of the hub genes and β-actin were synthesized by Sangon Biotech (Shanghai, China), and their sequences were listed in **Table 2**.

**Table 2:** Primer sequences used for quantitative Real-time PCR (qRT-PCR).

Gene symbol	Primer sequence
CDK1	F: 5′-AAACTACAGGTCAAGTGGTAGCC-3′ R: 5′-TCCTGCATAAGCACATCCTGA- 3′
CCNA2	F: 5′-GGATGGTAGTTTTGAGTCACCAC-3′ R: 5′-CACGAGGATAGCTCTCATACTGT- 3′
TOP2A	F: 5′-TTAATGCTGCGGACAACAAACA-3′ R: 5′-CGACCACCTGTCACTTTCTTTT- 3′
CCNB1	F: 5′-AATAAGGCGAAGATCAACATGGC-3′ R: 5′-TTTGTTACCAATGTCCCCAAGAG- 3′
KIF11	F: 5′-TGTTTGATGATCCCCGTAACAAG-3′ R: 5′-CTGAGTGGGAACGACTAGAGT- 3′
MELK	F: 5′-AACTCCAGCCTTATGCAGAAC-3′ R: 5′-AACGATTTGGCGTAGTGAGTATT- 3′
β-Actin	F: 5′-TTGATTTTGGAGGGATCTCGCTC-3′ R: 5′-GAGTCAACGGATTTGGTCGTATTG- 3′

### Statistical Analysis

Statistical analysis was performed through SPSS (version 23.0, Chicago, IL) and GraphPad Prism (version 6, San Diego, CA) software. Student’s *t*-tests were utilized for the comparison of two sample groups. Differences were considered as statistically significant when *P* < 0.05.

## Results

### Identification of DEGs

**Figure 1:**
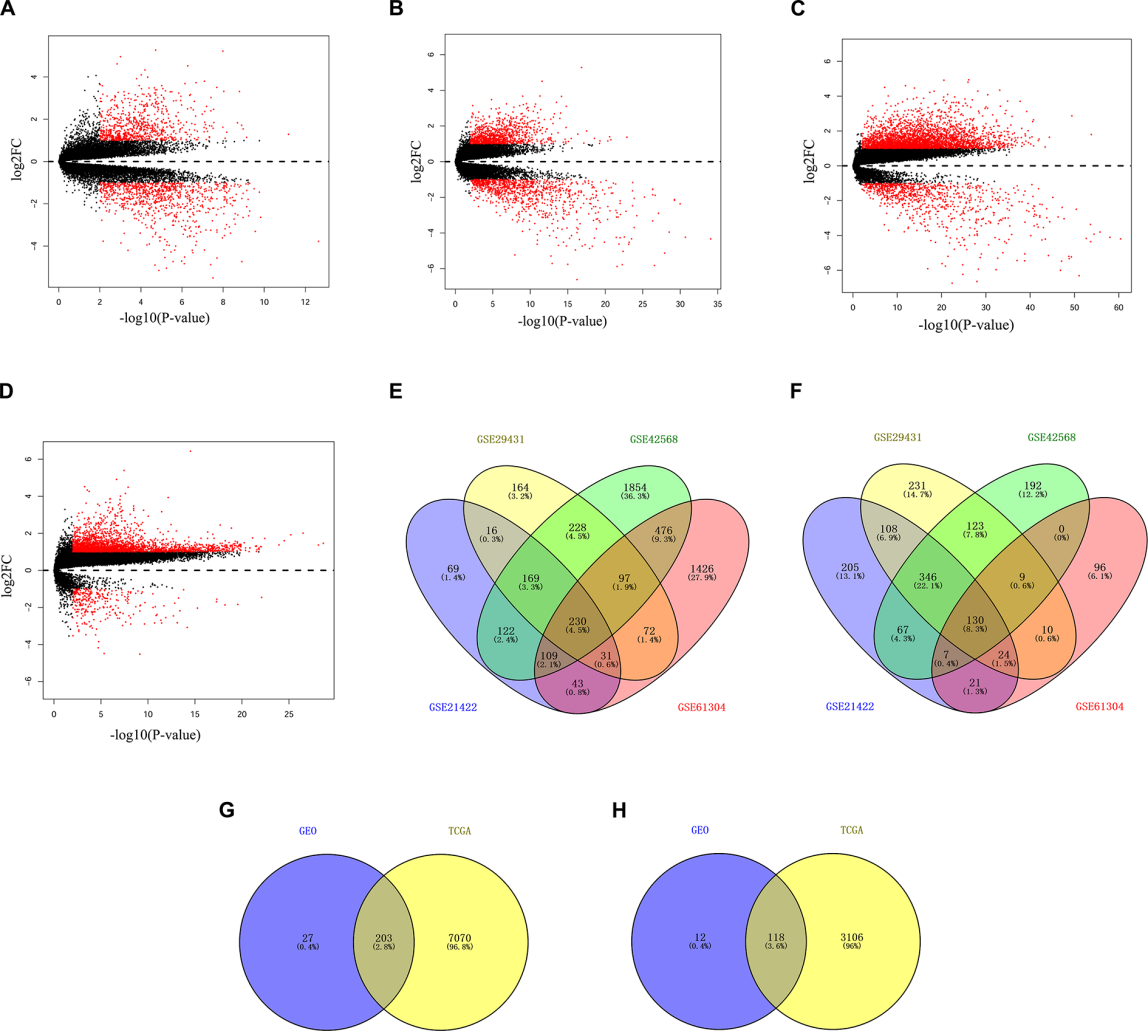
Identification of differentially expressed genes (DEGs) between breast malignant and non-malignant tissues. Panels **A**–**D** show the volcano plots of differentially expressed genes for dataset GSE21422 **(A)**, GSE29431 **(B)**, GSE42568 **(C)**, and GSE61304 **(D)**, respectively. Panels **E**–**F** show the Venn diagrams of the overlapping DEGs, including 230 up-regulated **(E)** and 130 down-regulated **(F)**, among the four datasets. Panels **G**–**H** show the Venn diagrams of a total of 321 DEGs, including 203 up-regulated **(E)** and 118 down-regulated **(F)**, among the four datasets of Gene Expression Omnibus (GEO) and the Cancer Genome Atlas (TCGA) datasets.

The discovery datasets (GSE21422, GSE29431, GSE42568, and GSE61304) were analyzed respectively to identify genes differentially expressed in breast non-cancerous and cancerous tissues. The discovery datasets included 38 non-cancerous breast tissue samples and 235 primary tumor samples obtained from multiple research sites (**Table 1**). There were 1,697 DEGs (789 up-regulated and 908 down-regulated) in GSE21422, 1988 DEGs (1,007 up-regulated and 981 down-regulated) in GSE29431, 4,159 DEGs (3,285 up-regulated and 874 down-regulated) in GSE42568, and 2,781 DEGs (2,484 up-regulated and 297 down-regulated) in GSE61304 which were differentially expressed between non-cancerous tissues and cancerous tissues as shown by volcano plots in **Figures 1A–D**. Further analysis of these DEGs by using Venn diagram revealed that there were 360 DEGs including 230 up-regulated and 130 down-regulated genes consistently observed in all four datasets (**Figures 1E, F**). To validate these DEGs, the breast cancer dataset (including 113 non-cancerous and 1,105 breast cancerous) from TCGA was downloaded and analyzed. A total of 321 DEGs including 203 up-regulated (**Figure 1G**) and 118 down-regulated genes (**Figure 1H**) identified in the discovery phase were confirmed in the TCGA dataset, resulting in an 89.2% consistency between the discovery and validation analysis. All 321 DEGs are listed in **Table 3**.

**Table 3:** Three hundred twenty-one differentially expressed genes (DEGs) were identified and confirmed from four profile datasets and the Cancer Genome Atlas (TCGA), including 203 up-regulated genes and 118 down-regulated genes in the breast cancer tissues, compared to normal breast tissues.

Regulation	DEGs (gene symbol)
Up-regulated	*S100P, COL10A1, RRM2, ADAMDEC1, UHRF1, KIF20A, GJB2, NUF2, PBK, RAB25, SPP1, DTL, SDC1, RAD51AP1, HOXC13, CKS2, UBE2C, EZH2, COL11A1, ESRP1, MMP1, SMIM22, KMO, TPX2, CEP55, MARCKSL1, CXCL10, BUB1B, FAM83D, CNTNAP2, HMMR, LMNB1, MELK, HIST1H2BD, TOP2A, KIF14, S100A14, GINS1, CDKN3, KRT8, NEK2, CXCL11, ANLN, TLCD1, CCNB1, SPAG1, TPD52, SLC44A4, SPAG5, PRC1, CDK1, EPN3, COMP, KIF11, CORO2A, SLC9A3R1, FOXM1, ZWINT, SPINT2, TRIM59, E2F5, KIF4A, TTK, HIST1H4H, NDC80, CDC7, DLGAP5, PYCR1, NUSAP1, PRSS8, SHCBP1, UBE2T, E2F8, CXCR4, PTK6, SLC12A8, MAD2L1, CCNE2, HIST1H2BH, ANKRD22, OIP5, CDC20, MNX1, HOXC10, CCNA2, TK1, ZNF367, MIF, MAL2, KIF15, CTHRC1, MCM10, ROGDI, LAMP5, UBE2S, ATAD2, CENPU, PTTG1, LRP8, TRIP13, WISP1, SULF1, AP1M2, STIL, CLDN7, NCAPG, KIF23, GGCT, ABRACL, KIF18B, SMYD3, AURKA, DEPDC1, SBK1, REM2, EVPL, CCDC167, KPNA2, TIGIT, CELSR3, PLEKHF2, PSRC1, INHBA, RMI2, C3orf80, FN1, CDCA3, NKX3-2, HMGB3, ERMP1, CENPM, FCGR1B, IL21R, C6orf99, SQLE, KIF2C, FANCI, CENPF, LLGL2, HELLS, MKI67, MMP11, RALGPS2, DDIAS, RACGAP1, ASF1B, EZR, RMI1, CAPG, MDK, ESPL1, PCNA, PLEK2, MCM2, CENPK, KNTC1,CDCA2, BARD1, POSTN, ASPM, CEMIP, PTTG3P, ZWILCH, RNF139-AS1, PLAUR, NME1, TYMS, KLHDC7B, NUP210, NEIL3, BGN, NVL, PKMYT1, MBOAT2, EDN2, ATP6V0B, ATP6AP1, POLQ, RGS4, RAD54L, LRRC15, ERCC6L, SMC4, BAIAP2L1, EXO1, SLAMF8, FEN1, SLC52A2, ICOS, PDIA4, VAMP8, HIST1H3E, ENTPD7, GPR84, CYB561, GPR19, RABIF, NOD2, MICAL2, MEX3A, FAM222A, CDCA8, ARSI*
Down-regulated	*DEFB132, LEP, PLIN1, CIDEC, ZBTB16, TIMP4, PLIN4, ACVR1C, LPL, GPAM, CFD, ITIH5, SLC19A3, LYVE1, CIDEA, AOC3, CHRDL1, FHL1, ADH1C, TNMD, KLB, MAOA, PPARG, TMEM100, C2orf40, ABCA6, CD36, FMO2, ACADL, GPR146, CA4, MRAP, PDK4, ATP1A2, MME, DMRT2, GDF10, NIPSNAP3B, SCN4B, IGFBP6, HLF, CAV1, FGF2, PCDH9, ADH1B, LRRN4CL, SLC16A7, SEMA3G, RBP7, EBF1, PPP1R14A, TSLP, TGFBR3, CORO2B, ANGPTL1, FIGN, BMP2, ANKRD29, ANXA1, COX7A1, ADAMTS5, PLAC9, PALMD, COL6A6, LINC00968, APCDD1, ASPA, SCARA5, IRS2, TMEM132C, CKMT2, SYNM, IGSF10, CCDC178, SRPX, GNG11, AKAP12, HOXA5, BTNL9, NRN1, MICU3, IL33, CFH, VIT, JAM2, ENPP2, PLSCR4, FOSB, PLAGL1, FAM149A, NR3C2, CREB5, MT1M, RERGL, MYOM1, ADRB2, PGM5-AS1, CLDN5, SPRY2, SEL1L2, ABCA9, RASSF9, FAM162B, EMCN, ITM2A, EDNRB, RBMS3, CASQ2, TSHZ2, GPC3, LHFP, SOX7, GGTA1P, ABCA8, INMT, CRYBG3, LRFN5, GSTM5*

### Functional Enrichment Analysis of DEGs

**Figure 2:**
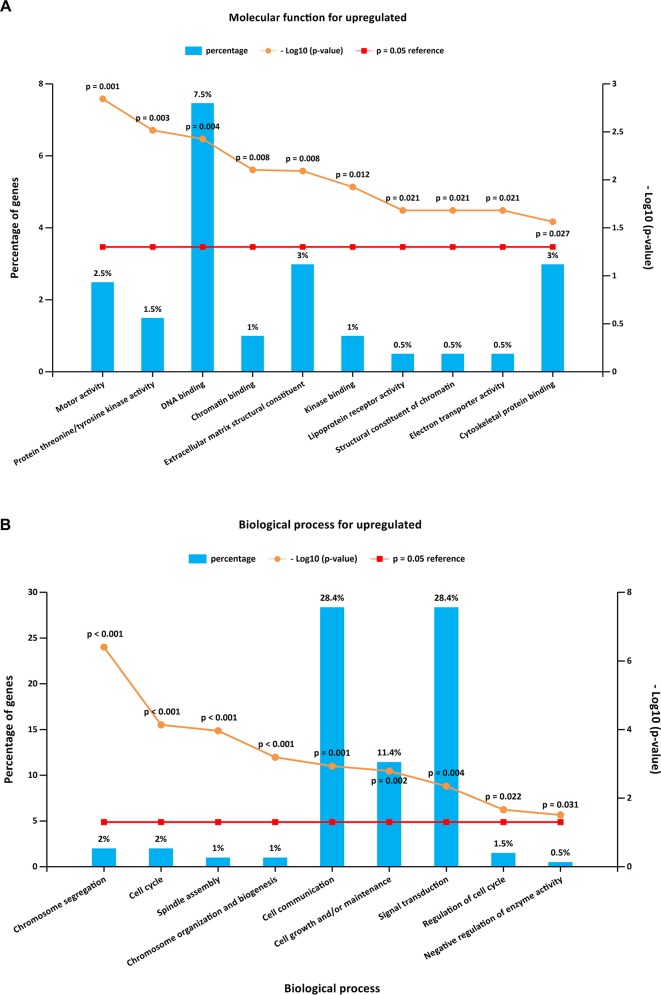
GO (Gene Ontology) enrichment analysis for upregulated DEGs. Panels **A**–**B** illustrate the top 10 elements significantly enriched in the GO categories: molecular function **(A)** and biological process **(B)**.

**Figure 3:**
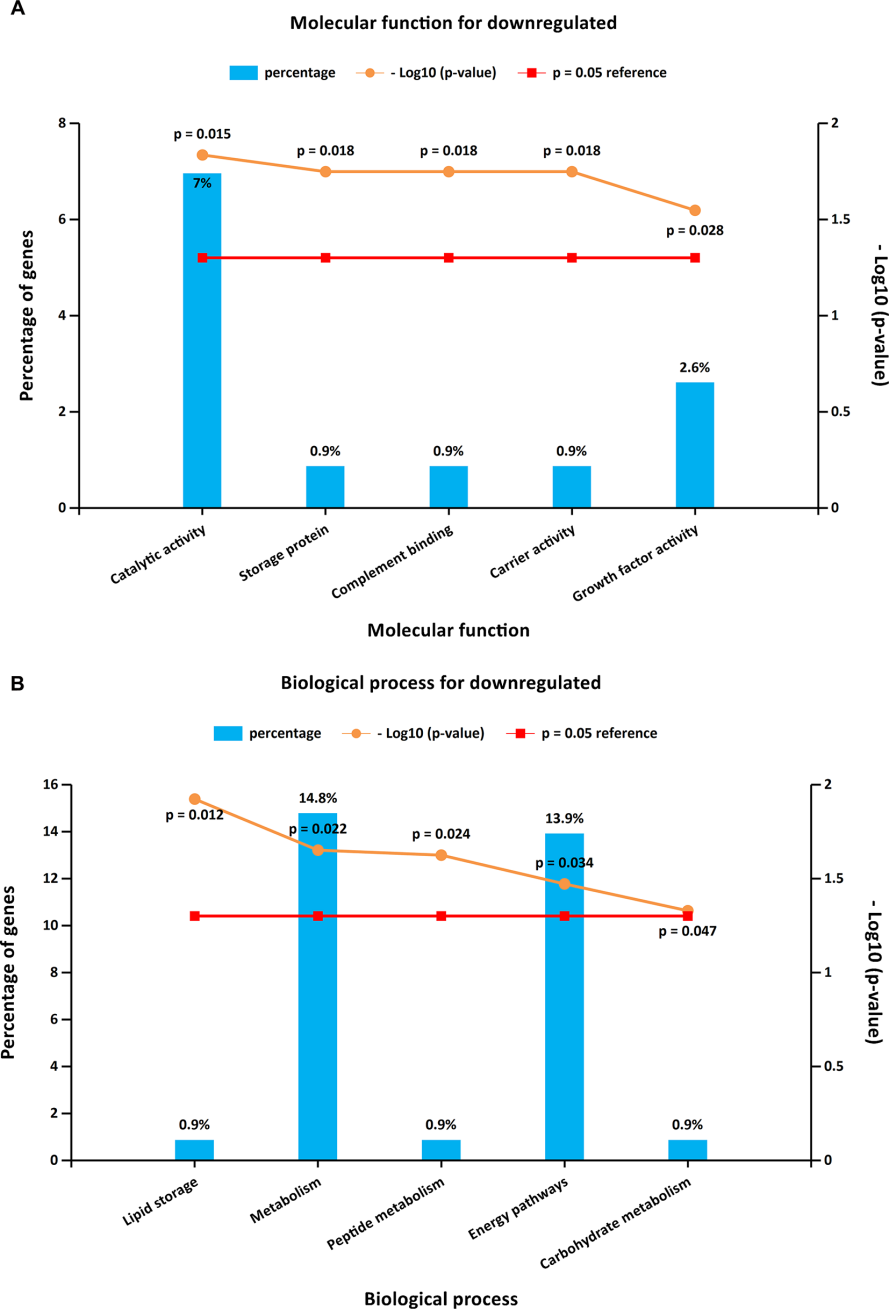
GO enrichment analysis for downregulated DEGs. Panels **A**–**B** illustrate the top 10 elements significantly enriched in the GO categories: molecular function **(A)** and biological process **(B)**.

To further investigate the biological functions of the 321 DEGs, GO analysis in FunRich was performed. The upregulated DEGs were mainly enriched in the motor activity, chromosome segregation, and cell cycle (**Figures 2A, B** and **Supplementary Table S1**), while the functional enrichment terms of downregulated DEGs were mainly correlated with the catalytic activity, lipid storage, and metabolism (**Figures 3A, B** and **Supplementary Table S1**).

**Figure 4:**
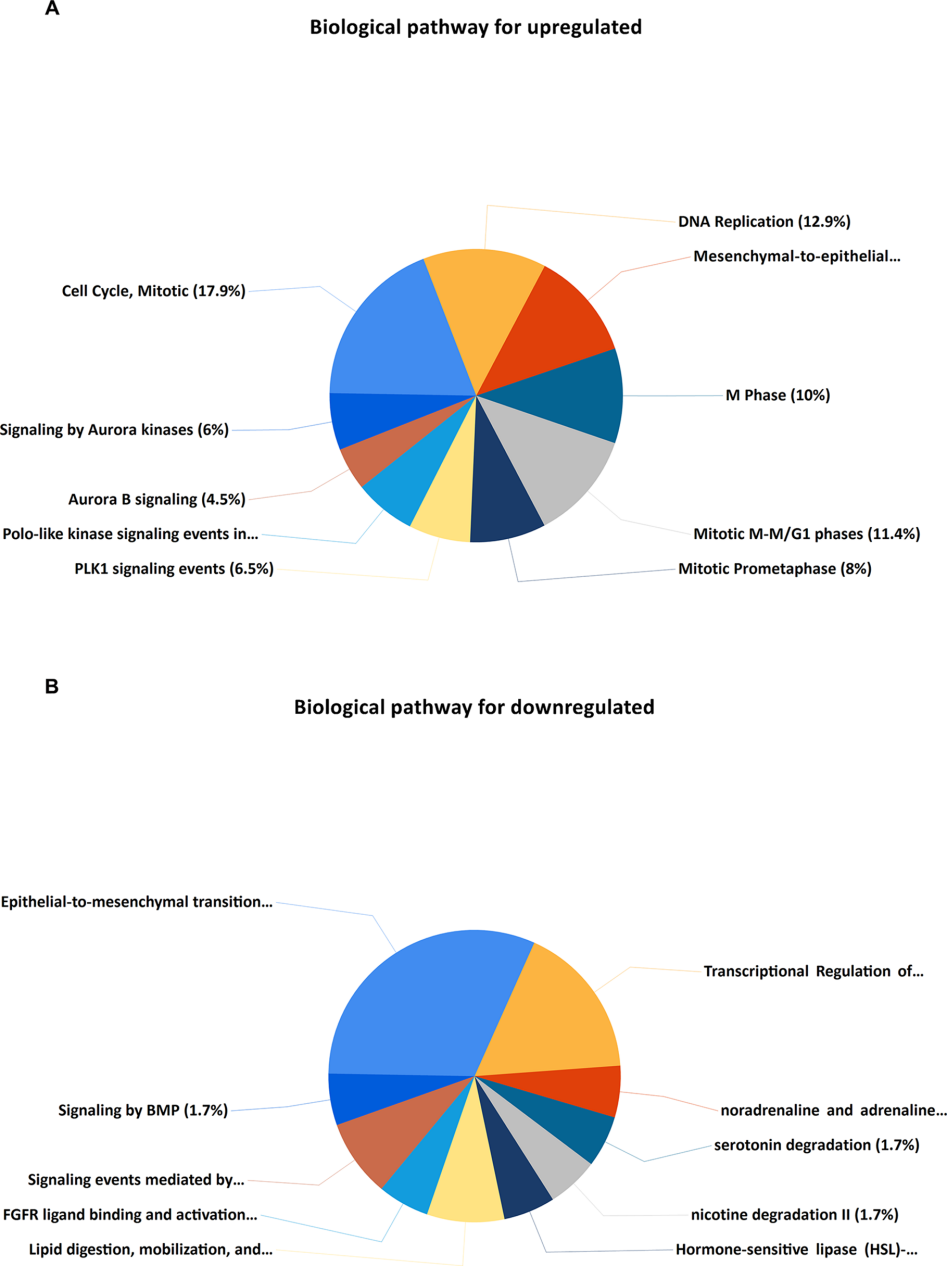
KEGG (Kyoto Encyclopedia of Genes and Genomes) pathway enrichment analysis of the DEGs. **(A)** Top 10 functional network/pathways associated with these upregulated DEGs through KEGG analysis with a *p* value less than 0.05. **(B)** Top 10 functional network/pathways associated with these downregulated DEGs through KEGG analysis with a *p* value less than 0.05.

Upregulated DEGs were particularly enriched in three pathways, including mitotic cell cycle, DNA replication, and mesenchymal-to-epithelial transition (**Figure 4A**). Furthermore, a vital gene CDK1 was significantly enriched in mitotic cell cycle pathway, DNA replication pathway, M phase pathway, mitotic M-M/G1 phase pathway, and PLK1 signaling event pathway (**Supplementary Table S1**). Two pathways that were notably enriched by downregulated DEGs were epithelial-to-mesenchymal transition, and transcriptional regulation of white adipocyte differentiation as shown in **Figure 4B**. However, a critical gene ANXA1 was significantly enriched in formyl peptide receptors bind formyl peptides and many other ligand pathway in biological pathway enrichment analysis for downregulated genes (**Supplementary Table S1**).

### PPI (Protein-Protein Interaction) Network and Module Analysis

**Figure 5:**
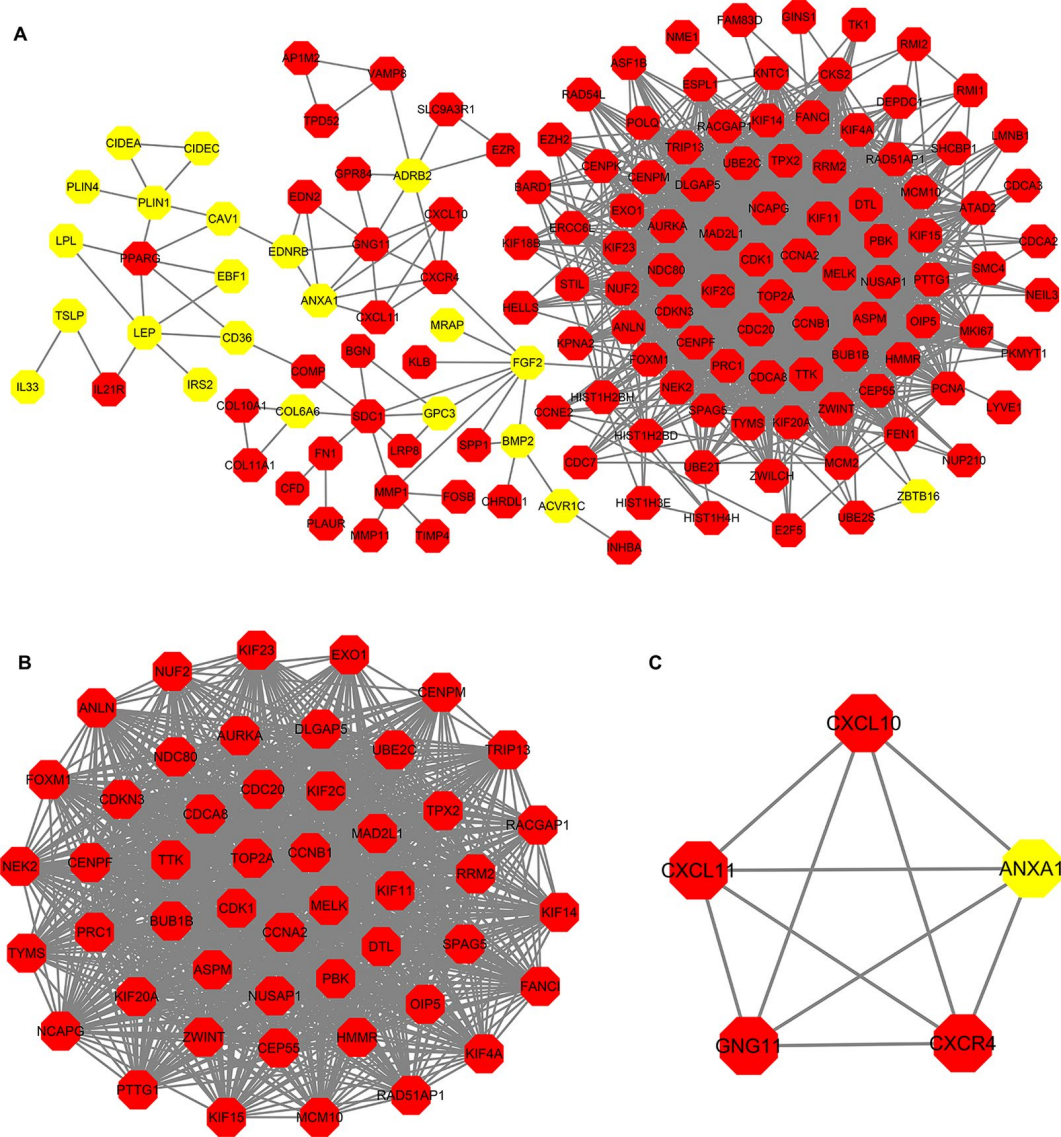
Protein-protein interaction (PPI) network construction. **(A)** PPI network constructed with the DEGs from the four datasets of GEO and TCGA datasets. **(B**–**C)** The significant module identified from the PPI network using the molecular complex detection (MCODE) method with a score of ≥ 5.0. Panel **B** shows the module 1 with an MCODE score of 46.63. Panel **C** shows the module 2 with an MCODE score of 5. The red nodes stand for upregulated genes, while the yellow nodes stand for downregulated genes.

**Figure 6:**
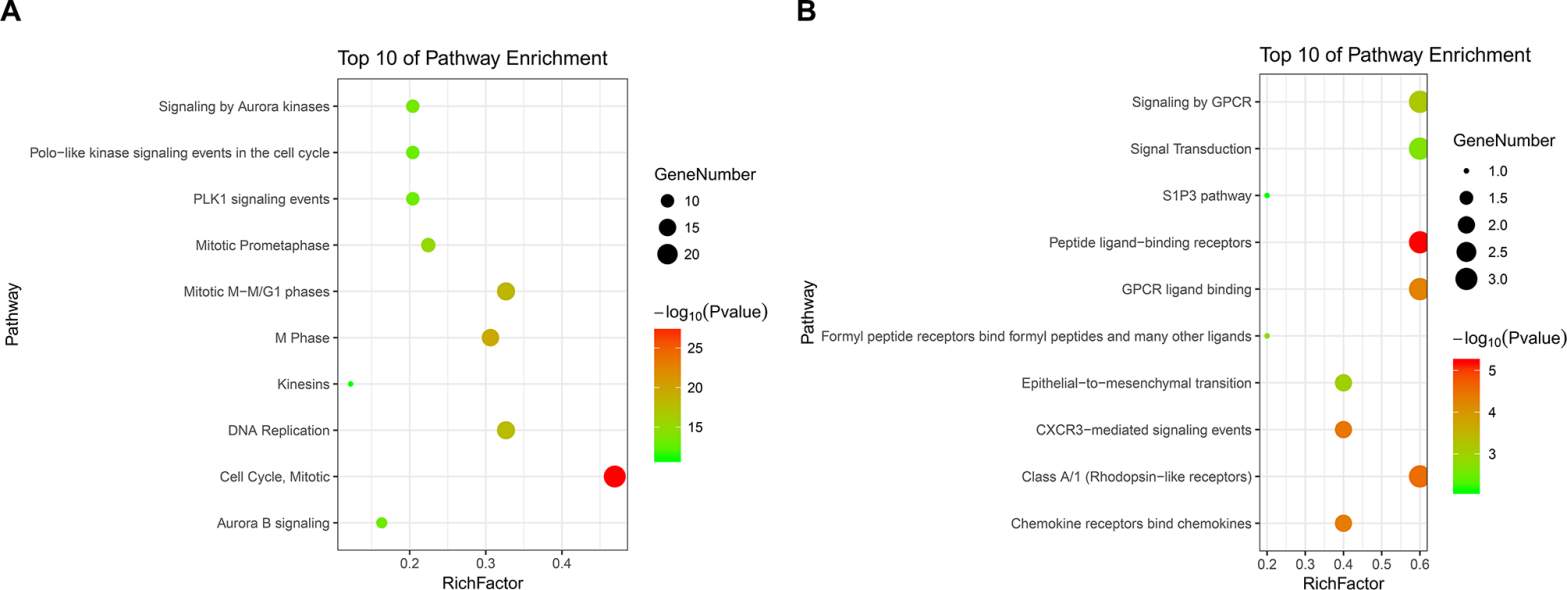
KEGG pathway enrichment analysis of module 1 and module 2. Panels **A** and **B** show the top 10 functional network/pathways associated with these genes in module 1 and module 2 through KEGG analysis, respectively, *P* < 0.05. Significantly enriched pathways of module 1 and module 2 are indicated in *Y*-axis. Rich factor in the *X*-axis represents the enrichment levels. The larger value of Rich factor represents the higher level of enrichment. The color of the dot stands for the different *P*-value and the size of the dot reflects the number of target genes enriched in the corresponding pathway.

PPI analysis of these DEGs revealed that there were 314 nodes and 1,810 interactions (**Figure 5A**). These proteins were selected based on a combined score ≥ 0.7 in STRING analysis. The vast majority of the nodes were upregulated DEGs in the network (**Figure 5A** and **Supplementary Table S2**). In addition, two significant modules (modules 1 and 2) with a score ≥ 5 were screened out *via* MCODE. CDK1, CCNA2, TOP2A, CCNB1, KIF11, and MELK were hub nodes with higher node degrees in module 1 (**Figure 5B**), and GNG11, ANXA1, CXCL11, CXCR4, and CXCL10 were hub nodes in module 2 (**Figure 5C**). Furthermore, six hub genes were selected for further analysis owing to the high degree of connectivity (**Table 4**). Enrichment pathways of module 1 and module 2 were displayed in **Figure 6** and **Supplementary Table S3**. The most significant pathway in module 1 and module 2 were enriched in the mitotic cell cycle pathway (**Figure 6A**) and the peptide ligand-binding receptors pathway **(** **Figure 6B**), respectively.

**Table 4:** Hub genes with high degree of connectivity.

Gene	Degree	Type	MCODE cluster
CDK1	78	UP	Cluster 1
CCNA2	72	UP	Cluster 1
TOP2A	68	UP	Cluster 1
CCNB1	68	UP	Cluster 1
KIF11	64	UP	Cluster 1
MELK	64	UP	Cluster 1

### Survival Analysis of Hub Genes

**Figure 7:**
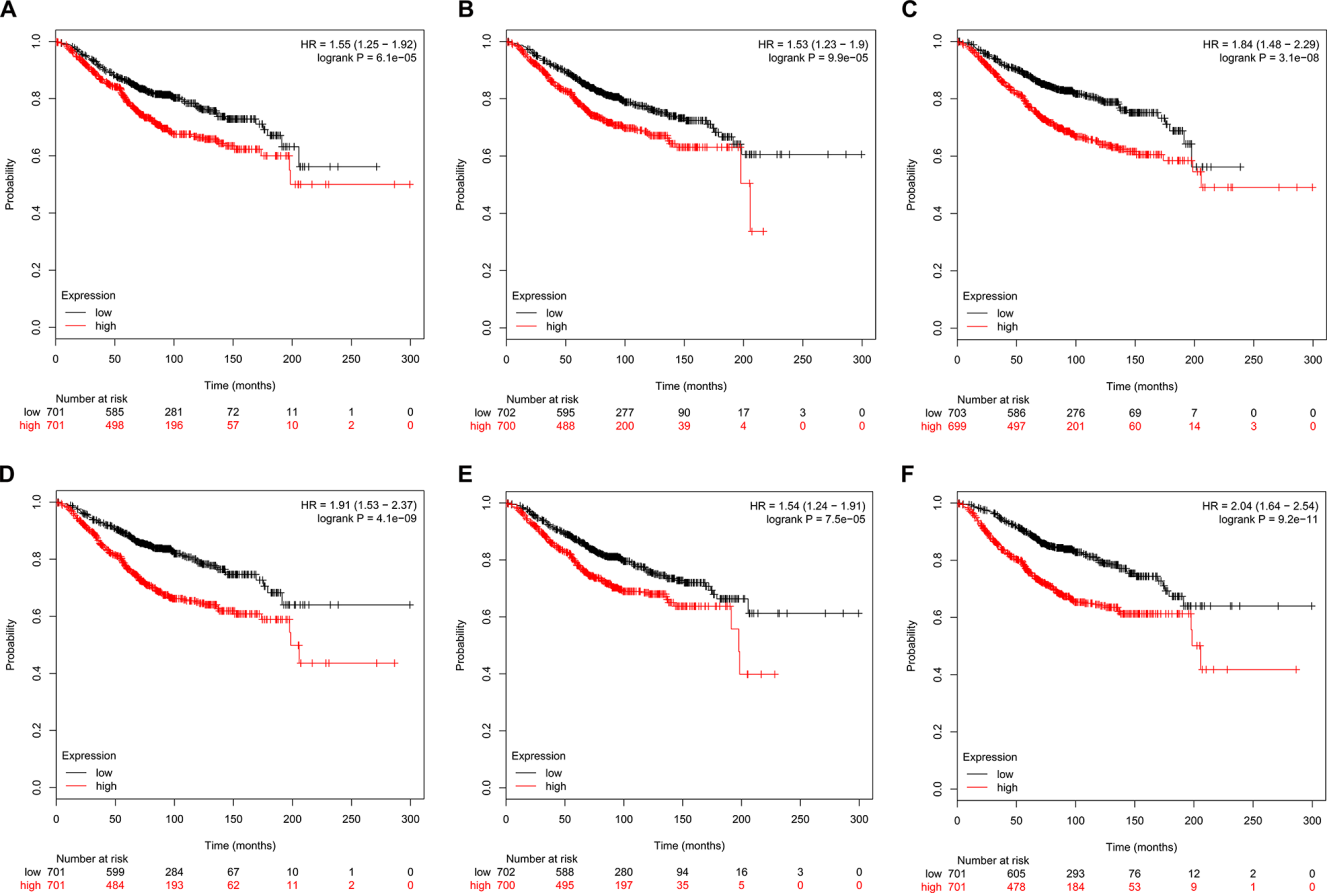
Kaplan–Meier survival curves of six hub genes in breast cancer patients. Overall survival (OS) by low and high **(A)** CDK1, **(B)** CCNA2, **(C)** TOP2A, **(D)** CCNB1, **(E)** KIF11, and **(F)** MELK expression.

The prognostic value of the six hub genes was explored in the website of Kaplan–Meier plotter. A high expression of CDK1 (HR 1.55 [1.25–1.92], *P* = 6.1e-05), CCNA2 (HR 1.53 [1.23–1.9], P = 9.9e-05), TOP2A (HR 1.84 [1.48–2.29], *P* = 3.1e-08]), CCNB1 (HR 1.91 [1.53–2.37], *P* = 4.1e-09), KIF11 (HR 1.54 [1.24–1.91], *P* = 7.5e-05]), and MELK (HR 2.04 [1.64–2.54], *P* = 9.2e-11) was related to a worse OS in breast cancer patients (**Figure 7**).

### Validation of Hub Genes Based on Multiple Clinic Pathological Features

**Figure 8:**
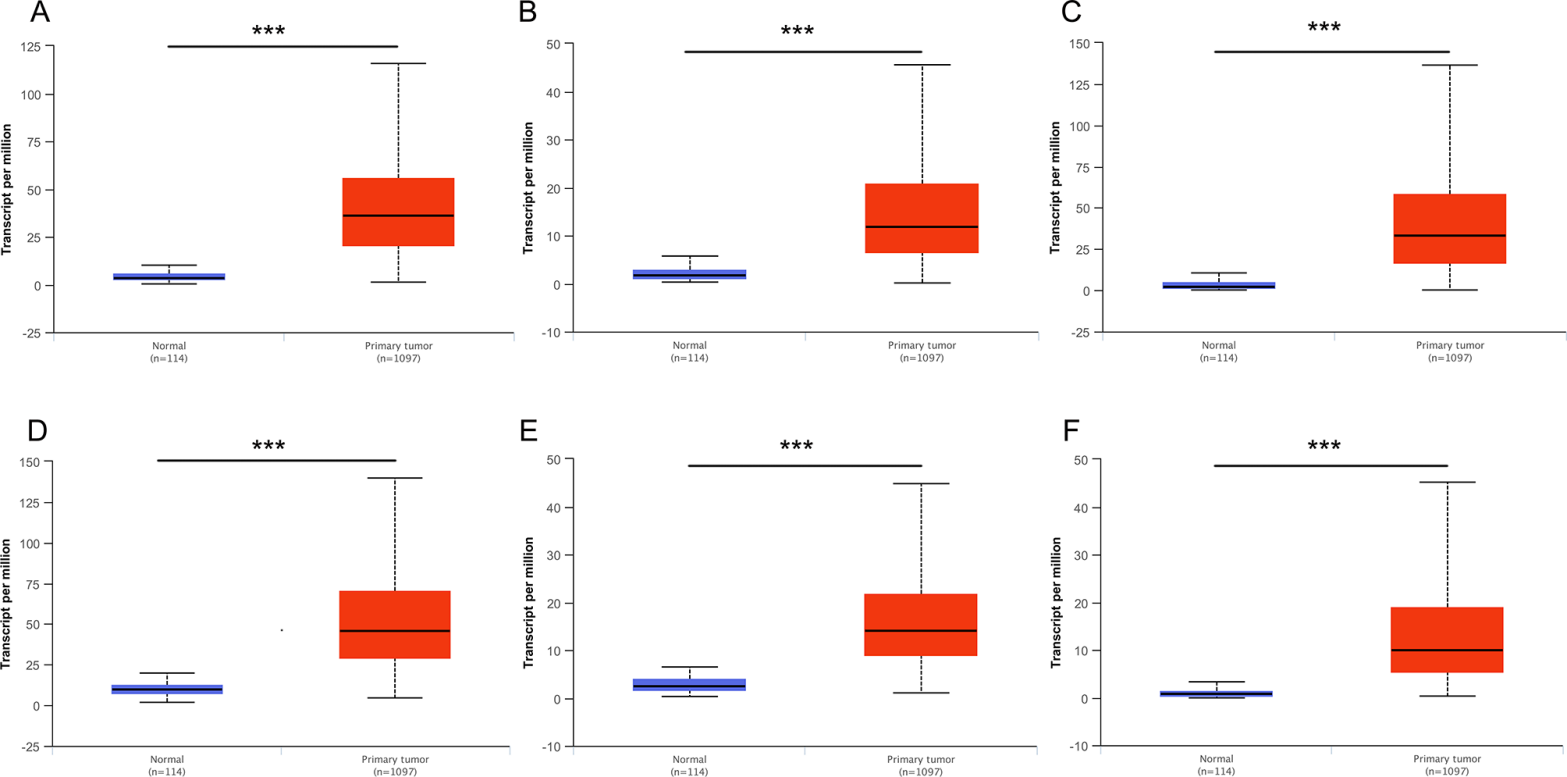
Relative expression of six hub genes in normal tissues and breast cancer tissues. **(A)** CDK1, **(B)** CCNA2, **(C) **TOP2A, **(D)** CCNB1, **(E)** KIF11, and **(F)** MELK. Data are mean ± SE. ***P < 0.001.

**Figure 9:**
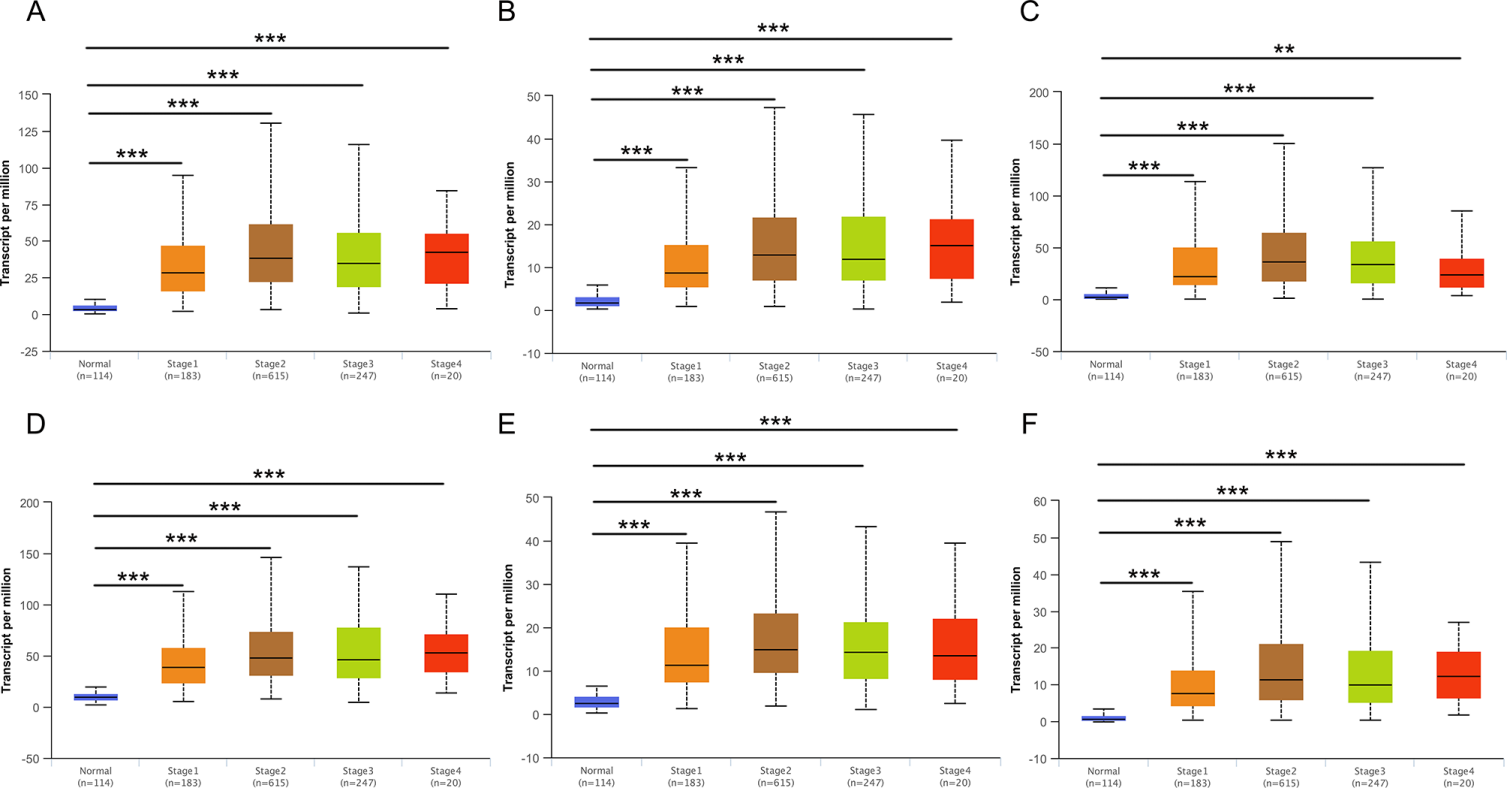
Relative expression of six hub genes in normal tissues and breast cancer tissues with different tumors stages. **(A)** CDK1, **(B)** CCNA2, **(C)** TOP2A, **(D)** CCNB1, **(E)** KIF11, and **(F)** MELK. Data are mean ± SE. **P < 0.01; ***P < 0.001.

**Figure 10:**
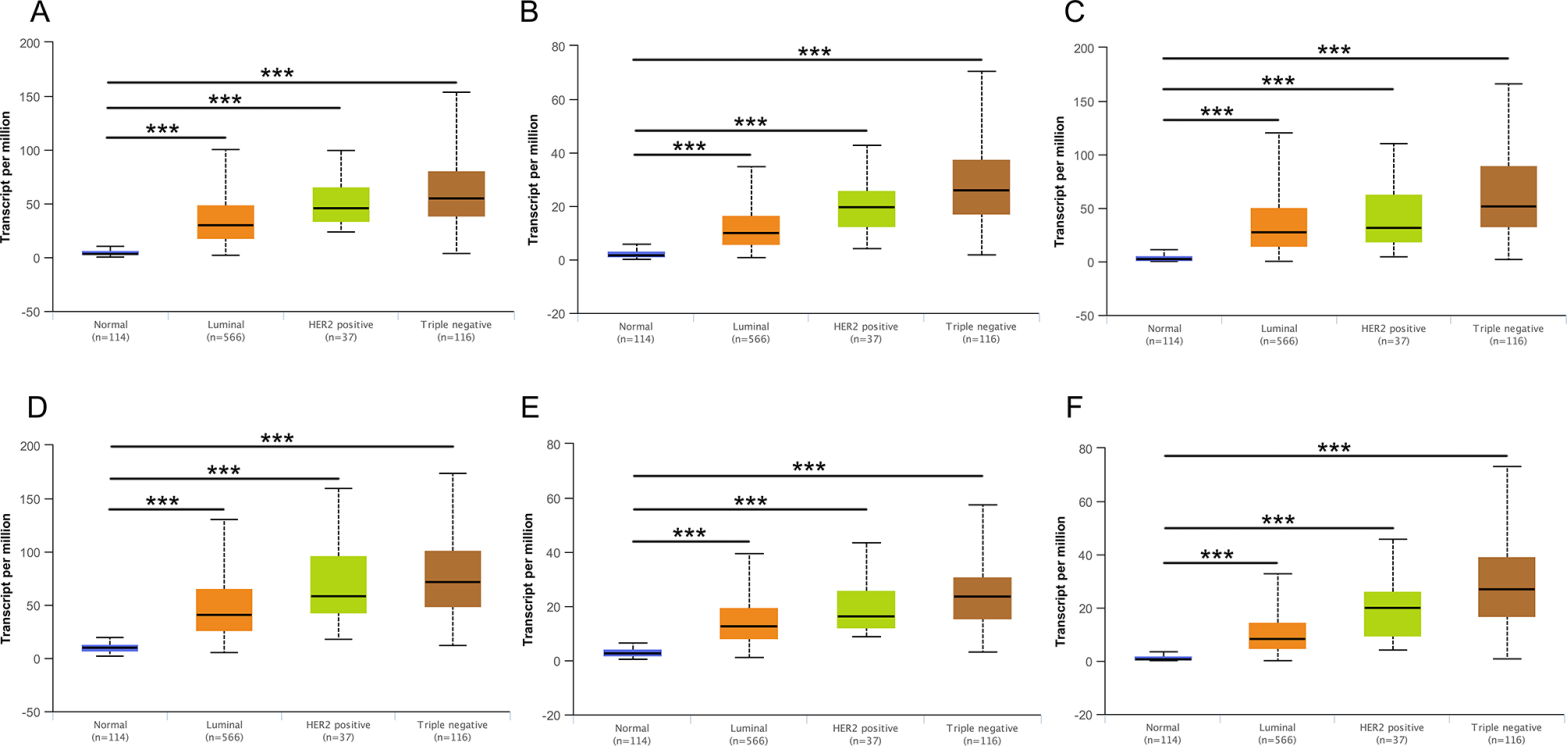
Relative expression of six hub genes in normal tissues and breast cancer tissues with different tumors subclasses. **(A)** CDK1, **(B)** CCNA2, **(C)** TOP2A, **(D)** CCNB1, **(E)** KIF11, and **(F)** MELK. Data are mean ± SE. ***P < 0.001.

**Figure 11:**
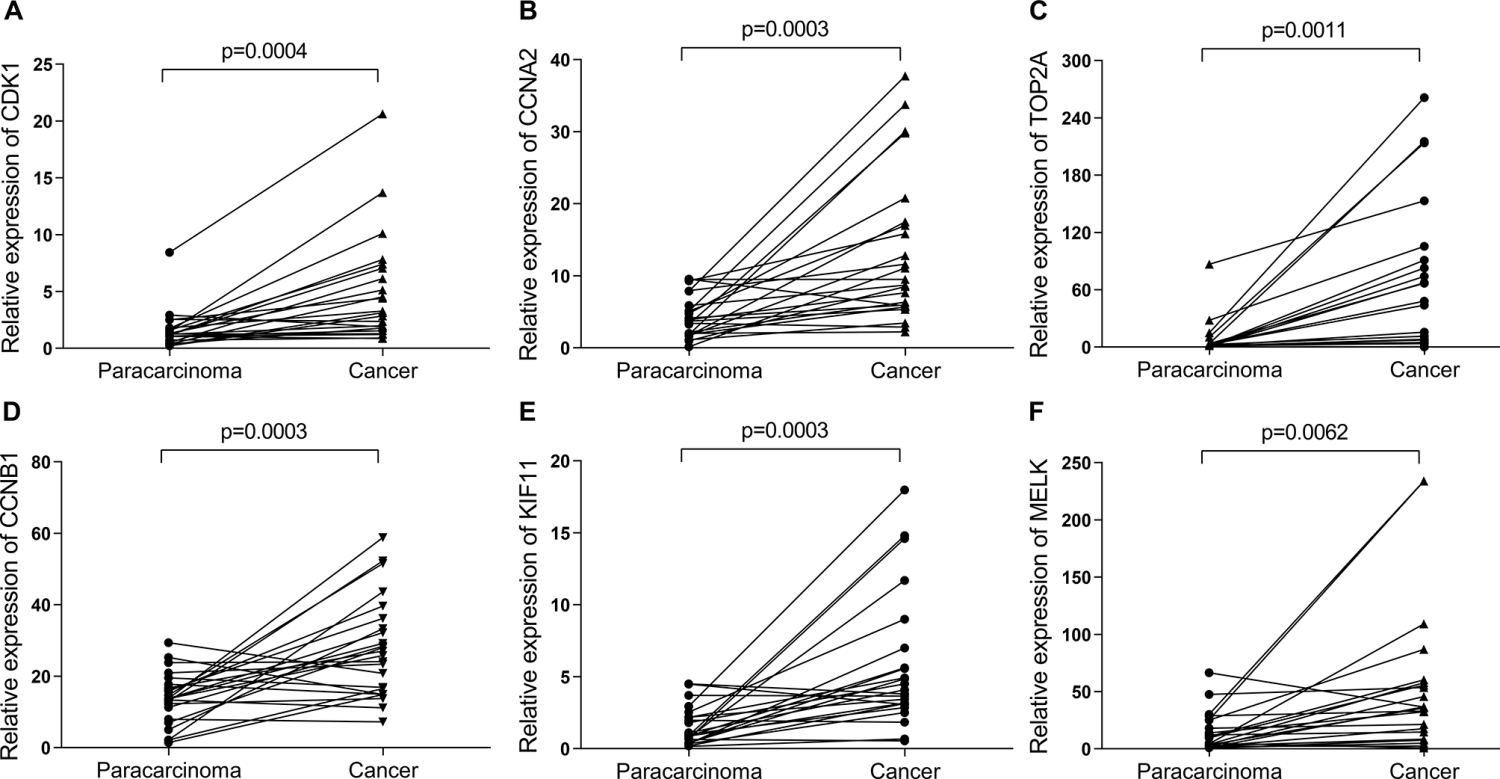
The relative expression of six hub genes in the breast cancer samples (*n* = 22) collected in our clinic were detected using quantitative Real-Time PCR (qRT-PCR). β-Actin was used as an internal reference gene for normalization. **(A)** CDK1, **(B)** CCNA2, **(C)** TOP2A, **(D)** CCNB1, **(E)** KIF11, and **(F)** MELK. Data were analyzed using paired Student’s *t*-test.

Then, UALCAN was applied to validate the expression levels of six hub genes in breast cancer (**Figures 8**–**10** and **Supplementary Figures 1**–**3**). The mRNA expression levels of six hub genes were all significantly higher in tumor tissues compared with those in normal tissues (**Figure 8**). Further subgroup analysis of multiple clinic pathological features of breast cancer samples in the TCGA consistently showed high-expression levels of six hub genes. The tumor stages and subclass boxplots of six hub genes were shown in **Figure 9** and **Figure 10**, respectively. The results demonstrated that expression levels of the hub genes were significantly associated with the stages and subclasses of tumors. In addition, the expression levels of these hub genes were significantly elevated in breast cancer samples than adjacent normal samples in subgroup analyses based on age, ethnicity, and menopause status of patients (**Supplementary Figures 1**–**3**). A significant overexpression of these six hub genes was also validated in the breast cancer samples (*n* = 22) collected in our clinic (**Figure 11**).

### Hub Gene-Drug Interaction Network Analysis

**Figure 12:**
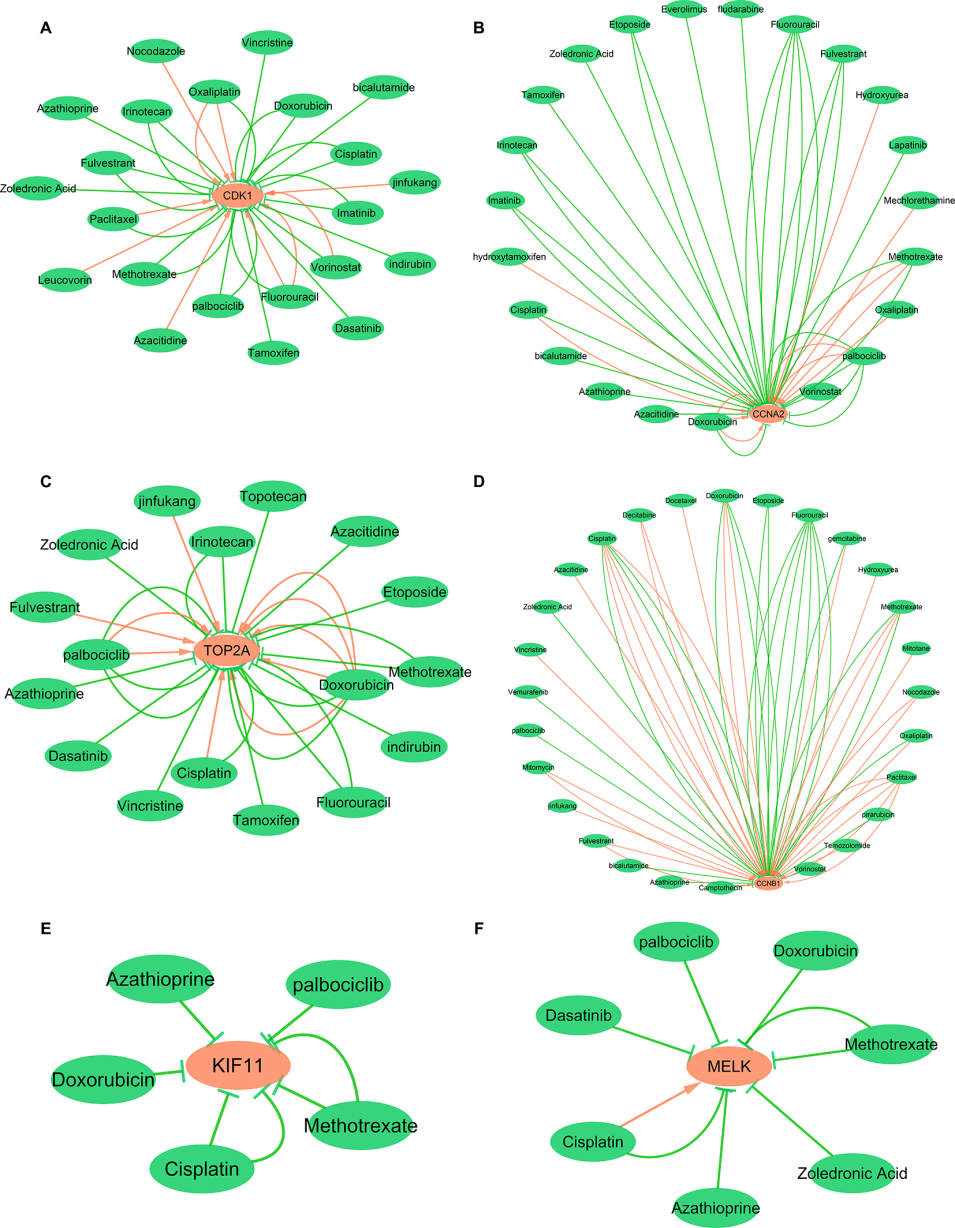
Gene-drug interaction network constructed with six hub genes and chemotherapeutic drugs. Panels **A**–**F** show available chemotherapeutic drugs decrease or increase the expression levels of hub genes in mRNA or protein. **(A)** CDK1, **(B)** CCNA2, **(C)** TOP2A, **(D)** CCNB1, **(E)** KIF11, and **(F)** MELK. Red arrows: chemotherapeutic drugs increase the expression of hub genes; green arrows: chemotherapeutic drugs decrease the expression of hub genes. The numbers of arrows between chemotherapeutic drugs and hub genes in this network represent the supported numbers of literatures by previous reports.

To explore the interaction between hub genes and available therapeutic drugs of cancer, the hub gene-drug interaction network was constructed using CTD and visualized by Cytoscape. As shown in **Figure 12**, a variety of drugs could affect the expression of these six hub genes, CDK1, CCNA2, TOP2A, CCNB1, KIF11, and MELK. For example, tamoxifen and doxorubicin could reduce CDK1 expression level while azacitidine could elevate CDK1 expression level (**Figure 12A**).

## Discussion

In the current study, we have gained insight into gene expression modules in breast cancer at a genome-wide scale through analyzing multiple breast cancer datasets. A panel of 321 DEGs (**Figures 1G, H**, **Table 3**) and six hub genes (see **Table 4**) have been identified, which are associated with breast cancer tumorigenesis and progression. These six hub genes were overexpressed in the breast cancer tissues compared to normal and non-cancerous tissues (**Figures 8**–**11** and **Supplementary Figures 1**–**3**), and the overexpression of each hub gene was associated with poor overall survival in breast cancer patients (**Figure 7**), suggesting that these hub genes may have “driver” function in breast cancer progression.

Among the 321 identified DEGs, notable dysregulation of gene expression was observed in mitotic cell cycle, DNA replication, and mesenchymal-to-epithelial transition. Cell cycle is an evolutionarily conserved process and essential for cell growth. Dysfunctions of cell cycle are a hallmark of human cancer (Dominguez-Brauer et al., 2015). Numerous therapeutic strategies have been implemented to target cell cycle in the treatment of cancer. Accumulating studies have demonstrated that several cell cycle–related genes such as CCNB1, CCNA2, and CDK1, which were also identified in the current study, are involved in the initiation and development of cancer. Ding et al. (2014) have demonstrated that CCNB1 could be a biomarker for the prognosis of patient with ER-positive breast cancer and for the monitoring of hormone therapy efficacy. Shi et al. have recently reported that ISL1-induced cell proliferation and tumorigenesis in gastric cancer were mediated through the regulation of CCNB1, CCNB2, and C-MYC expressions (Shi et al., 2016). These reports are in agreement with our current demonstration that CCNB1, as a hub gene, was overexpressed in breast cancer tissues, and its overexpression was associated with poor patient outcome.

Previous reports revealed that cell cycle and apoptosis are two major dysregulated events in cancer cells (Evan and Vousden, 2001). Cyclin-dependent kinases (CDKs) are proved to be important proteins in the regulation of cell cycle (Malumbres and Barbacid, 2009). An inhibition of CDK1 is able to suppress tumor growth and induce apoptosis in triple-negative breast cancer (Liu et al., 2014). Moreover, Kim et al. reported that breast cancer patients with specific high activity of CDK1 and CDK2 had significantly poorer 5 years of relapse-free survival compared to those with low CDK1 and CDK2 activity (Kim et al., 2008), similar to our current observation (**Figure 7**). Similar association has also been reported in renal cell carcinoma (Hongo et al., 2014). Taken together, these data suggest that CDK1 and CDK2 may serve as potential biomarkers for predicting the outcome of cancer patients, especially those with breast cancer.

CCNA2 functions as a key regulator of cell cycle and is reported to be up-regulated in many cancers including breast cancer. Gao et al. (2014) have found that a high expression of CCNA2 in ER+ breast cancer patients was associated with a poor outcome in overall survival (OS), disease-free survival (DFS), recurrence-free survival (RFS), and distant metastasis–free survival (DMFS), similar to our current finding (**Figure 7**). In addition, overexpression of CCNA2 was closely associated with a poor efficacy of tamoxifen therapy in ER+ breast cancer patients (Gao et al., 2014). Recently, Gan et al. have revealed that a high expression of CCNA2 was observed in colorectal cancer tissues, and knockdown of CCNA2 could impair cell cycle progression and induce cell apoptosis (Gan et al., 2018). Taken together, these data suggest that CCNA2 may be a prognostic biomarker for ER+ breast cancer and tamoxifen resistance.

Unlike CCNB1, CCNA2, and CDK1, currently, the remaining genes (TOP2A, KIF11, and MELK) are not found to be associated with cell cycle; their dysfunctions might still affect the progression and prognosis of breast cancer. TOP2A was a significant prognostic factor in predicting the breast cancer patient OS, and low expression of TOP2A was associated with a better clinical outcome (Xu et al., 2015). In addition, like HER2 amplification, TOP2A amplification or deletion was associated with an increase in responsiveness to anthracycline-containing chemotherapy regimens relative to non-anthracycline regimens (O’Malley et al., 2009). These reports collectively illustrate that TOP2A may be a prognostic factor for breast cancer and implicated a role on anthracycline-containing chemotherapy regimens.

Several articles have elucidated the functions of KIF11 in breast cancers—for example, Pei et al. demonstrated that KIF11 was upregulated in 95.8% paraffin-embedded archival breast cancer biopsies, and decreased expression of KIF11 inhibited the proliferation of breast cancer cells *in vitro* and *in vivo* (Pei et al., 2017). Besides, Zhou et al. found that the inhibition of KIF11 significantly decreased the cell viability, colony formation, as well as migration and invasion, but promoted apoptosis (Wang et al., 2014). These data conclude KIF11 as a potential oncogene that regulates the development and progression of breast cancer.

However, there were several contradictory studies that explored the function of MELK in basal-like breast cancer. Wang et al. elucidated that MELK was highly overexpressed in breast cancer, and its overexpression was strongly correlated with poor prognosis. Functionally, the ablation of MELK selectively inhibited the proliferation of basal-like breast cancer, but not type of luminal breast cancer cells both *in vitro* and *in vivo* (Wang et al., 2014). Nevertheless, recently, Huang et al. demonstrated that MELK was not required for the proliferation of basal-like breast cancer cells (Huang et al., 2017), to some extent, which was consistent with another study conducted by Giuliano et al. (2018). Taken together, these data suggest a confused role of MELK in basal-like breast cancer and further study is required.

To further explore the possibility of these hub genes as potential therapeutic targets for breast cancer, we analyzed the interaction between six hub genes and available therapeutic drugs of cancer and found that numbers of drugs could affect the expression of these hub genes. Nevertheless, whether breast cancer patient with overexpression of these hub genes could benefit from the suppression of hub genes, or whether these hub genes are promising, therapeutic targets still need further experimental supports including pre-clinical and prospective clinical studies.

In current, some relevant studies based on the database were published regarding hub genes in breast cancer. For instance, Fang et al. identified 15 genes from one GEO datasets by bioinformatic approach including the raw data analysis of GSE10797, GO and KEGG pathway enrichment, PPI network construction and module analysis, survival analysis of hub genes, and Connectivity Map (cMap) database analysis (Fang and Zhang, 2017). Tang et al. identified 10 hub genes in brain metastasis breast cancer from two GEO databases, and four hub gene expression of which were closely associated with the OS of breast cancer patients by developing an integrated method including GO and KEGG pathway enrichment analysis, PPI network analysis, hub gene identification, transcription factor (TF) analyses, and OS analysis (Tang et al., 2019). Chen et al. identified NCAPG and ABCA9 as key genes in TNBC by weighted gene co-expression network analysis (Chen et al., 2019). Compared to their findings, the hub genes we identified in current study are not exactly consistent with their results. The different datasets and analysis methods were utilized in our study, which might partly account for the reasons for these differences. Some advantages of our study mainly lie in the following points: first of all, this study integrates data with relative larger sample size from multiple GEO datasets and TCGA datasets. Secondly, this study validates the differential expression of six hub genes in the breast cancer samples collected in our clinic, to some extent, which partly suggests the reliability of integrated bioinformatic analysis. Thirdly, this study establishes gene networks and identifies potential diagnostic and prognostic biomarkers in breast cancer. Fourthly, this study considers the effects of traditional clinicopathological prognostic factors on the expression of hub genes such as age, races, tumors stages, as well as subclass of breast cancer. Finally, the exploration of interaction between six hub genes and available therapeutic drugs of cancer may provide some potential help for further finding new biomarkers and targets for drug synthesis of breast cancer.

In current study, we have discussed that high expression of six hub genes is involved in the development of breast cancer and associated with worse OS, suggesting that these hub genes may serve as potential prognostic biomarkers and therapeutic targets for breast cancer. However, the limitations of our study also should be recognized. First of all, when analyzing the DEGs, in view of the complexity of datasets in our study, it is difficult to consider some important factors—for example, different age, races, regions, as well as tumor staging and classification of patient. Secondly, according to the results, the six hub genes were all up-regulated in breast cancer, but the mechanism of up-regulation was not clear. Therefore, more evidences are required to find out the biological foundation. Finally, this study mainly focuses on analyzing the expression levels and OS of six hub genes, while whether these hub genes could be used as biomarkers or could improve the diagnostic accuracy and specificity for breast cancer need further study.

## Conclusion

Based on integrated bioinformatic analysis, the present study has identified 321 DEGs and six hub genes (CDK1, CCNA2, TOP2A, CCNB1, KIF11, and MELK) that are associated with breast cancer tumorigenesis and progression. The overexpression of each of these six hub genes in breast cancer as demonstrated in database analysis and confirmed in prospective analysis of breast cancer samples indicates a poor clinical outcome in breast cancer patients. These results collectively suggest that a comprehensive investigation of these DEGs will facilitate our understanding of breast cancer pathogenesis and progression. Furthermore, these hub genes may serve as potential prognostic biomarkers and therapeutic targets for breast cancer, which remains to be validated by further pre-clinical and prospective clinical studies.

## Data Availability

Publicly available datasets were analyzed in this study. This data can be found here: https://www.ncbi.nlm.nih.gov/gds/

## Author Contributions

J-LD conceived, designed the research, conducted the experiments, analyzed the data and wrote the manuscript. Y-HX participated in the collection of clinical samples. GW participated in the experimental design and provided financial and instrumental support. All authors read and approved the final manuscript.

## Funding

This work was supported by the National Natural Science Foundation of China (grant no. 81673516), the Fundamental Research Funds for the Central Universities of Central South University (grant no. 2019zzts343) and internal funding from Central South University in China.

## Conflict of Interest Statement

The authors declare that the research was conducted in the absence of any commercial or financial relationships that could be construed as a potential conflict of interest.
