# Supplementary figures and images for: Identification of Potential Crucial Genes and Key Pathways in Breast Cancer Using Bioinformatic Analysis
Source: Front Genet. 2019 Aug 2;10:695. doi: 10.3389/fgene.2019.00695 (PMC6688090; doi:10.3389/fgene.2019.00695)

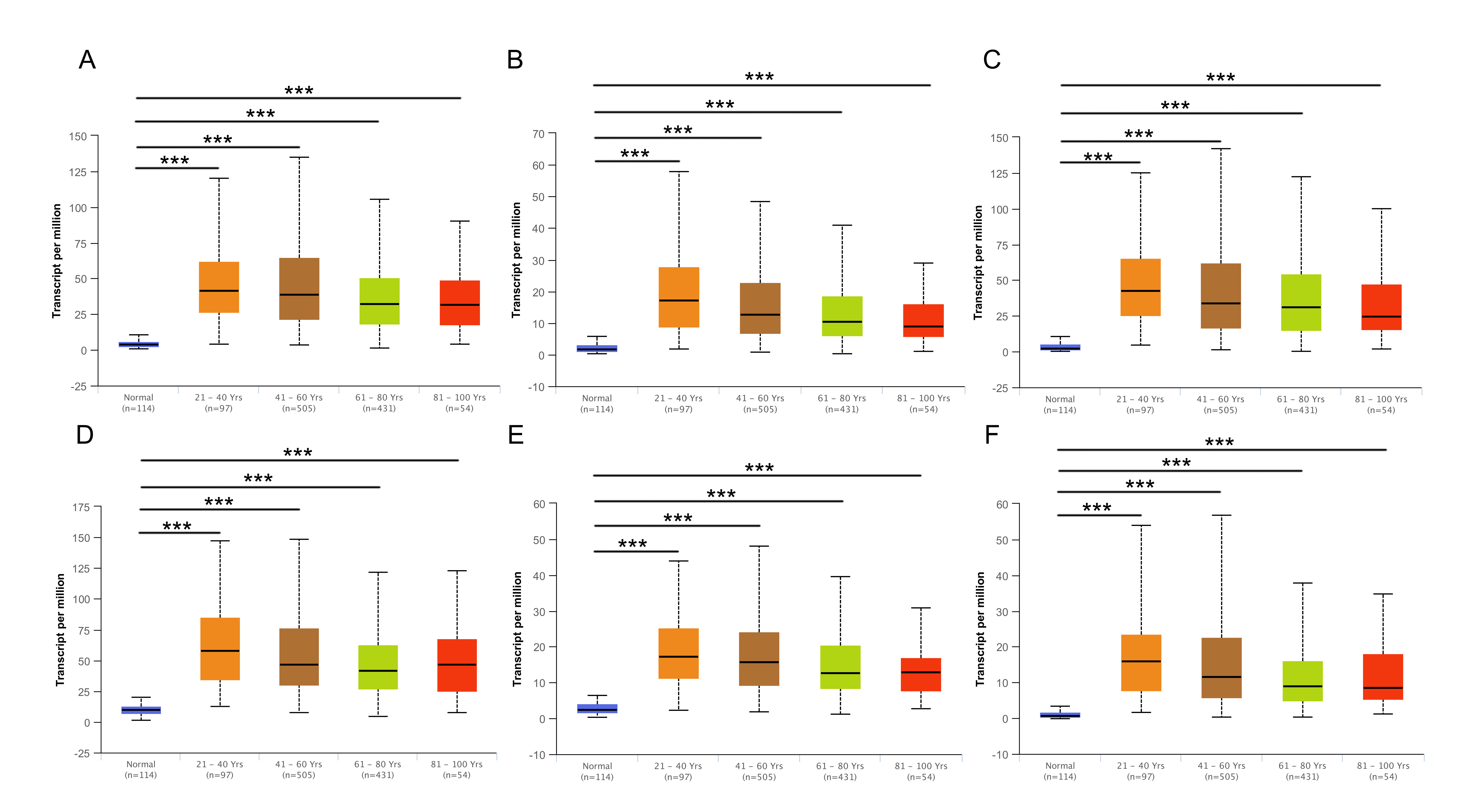

Supplement: Supplementary file 1 [file Image_1.tif]

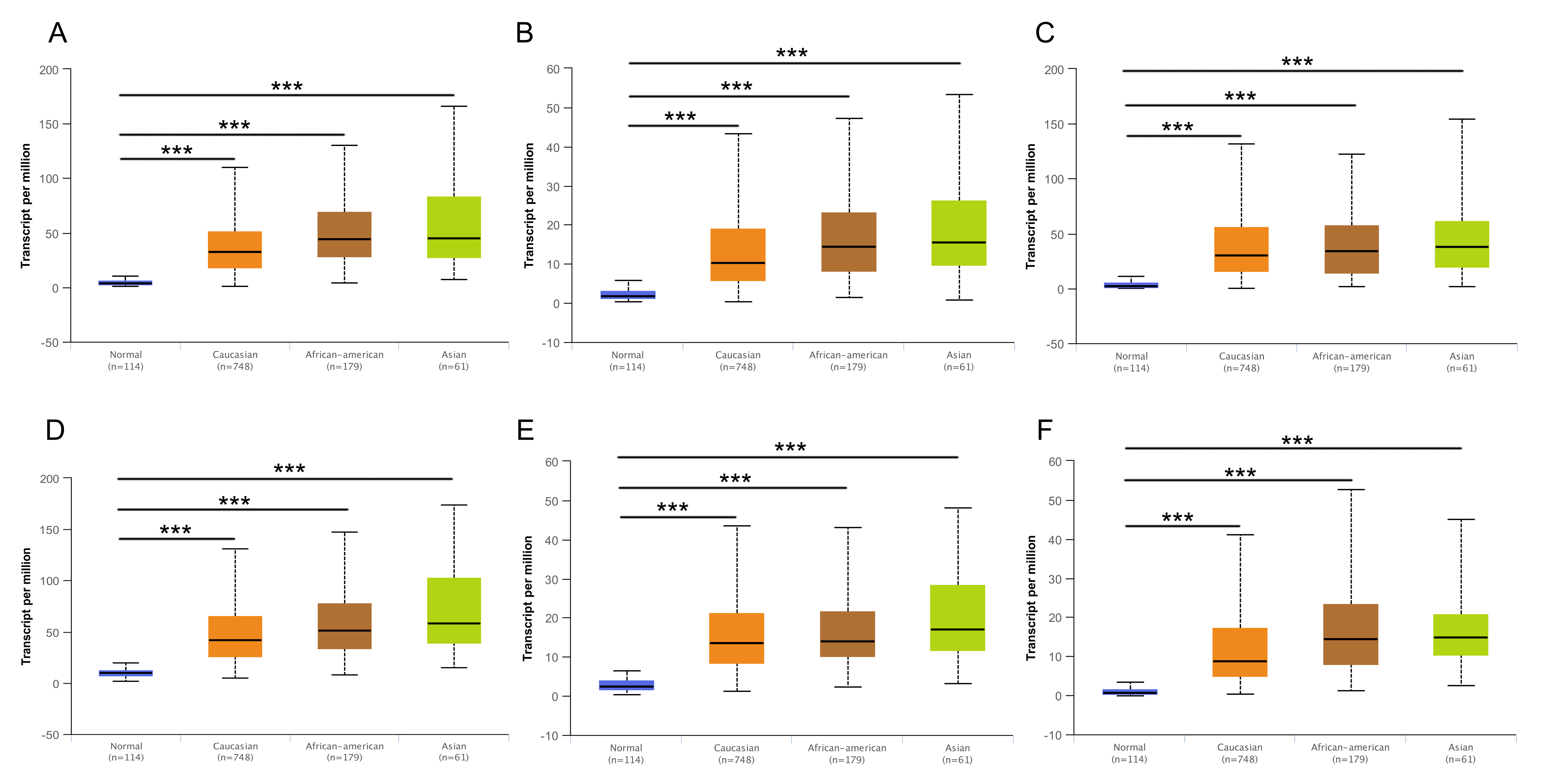

Supplement: Supplementary file 2 [file Image_2.tif]

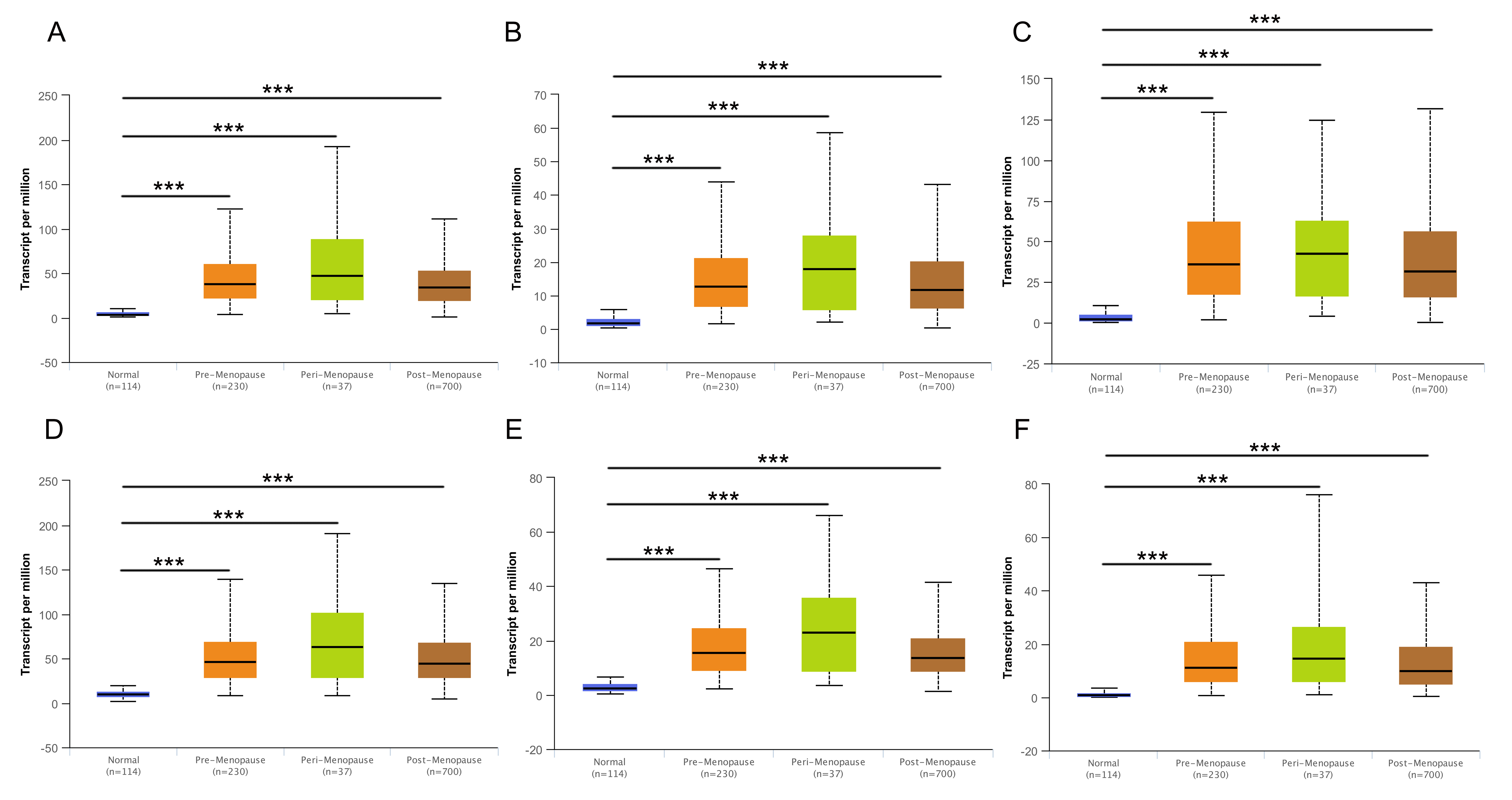

Supplement: Supplementary file 3 [file Image_3.tif]
